# Diseases of the Lungs, Heart, and Kidneys

**Published:** 1893-04

**Authors:** 


### BOOK NOTICES.

Diseases of the Lungs, Heart, and Kidneys. No. 14 in the Physicians and Students' Ready Reference Series. By N. S. Davis, Jr., A. M., M. D. Published by the F. A. Davis Co.: Philadelphia (1231 Filbert Street) and London. Price, $2.50.

This book is divided into three sections, as its name indicates. The first treats of diseases of the Bronchi, Lungs, and Pleura; the second of Diseases of the Heart, and the third of Diseases of the Kidneys. Each section has its chapters, which are devoted to the diseases of these organs. The subjects are clearly and concisely described. Chapter XIII, on Pulmonary Tuberculosis, contains forty-six pages on this affection, which should be well studied, as this fell disease is one that is the greatest scourge to mankind in general. As stated above in the title, this work forms another one of Davis & Co.'s Ready Reference Series, so many of which have been reviewed and cordially commended in the pages of this journal.

